# The Incorporated Society of Trained Masseuses

**Published:** 1916-05-13

**Authors:** 


### THE INCORPORATED SOCIETY OF TRAINED MASSEUSES.

### ITS APRIL EXAMINATIONS.

An examination of the Society of Trained Masseuses took place on Friday and Saturday, April 7 and 8. The candidates, who numbered 113, had presented themselves at a written examination on theory of massage and anatomy and physiology, held under the auspices of the Society, either in London or some local centre—Liverpool, Birmingham, Bradford, Bristol, etc.—some weeks previously, and on April 7 they were examined orally by a woman doctor. But the great ordeal was on April 8, when the candidates underwent the practical examination, which took place at the Examination Hall, Queen Square, W.C.

Thirty-six examiners and thirty-four models were at work on April 8 in a series of eighteen improvised rooms, each containing a couch on which a model was to be seen dressed in marvellously fashioned garments abundantly furnished with tapes and buttons to allow of massage operations being performed without inconvenience. Pillows and blankets were also liberally supplied. The candidate is expected to make use of the former to give support to the parts receiving treatment, the latter to demonstrate the best methods of keeping the patient warm whilst undergoing treatment. Having been conducted from one examiner to another until her ordeal is over the candidate at last finds herself back in the waiting-room from which she started an hour earlier.

Miss Holberton, the director of the examinations, is responsible for the work of arranging for examiners, models, candidates, and stewards keeping to the time-tables with mechanical exactitude. The examiners, who are all successful massage teachers, work in periods of two or three hours, and great care is taken that no candidate is personally known to the examiners before whom she will appear.

Candidates for this examination must have received a minimum of six months' training, with an average of at least twelve hours' instruction a week, exclusive of work on patients. If the instruction is spread over a longer time, a proportionate reduction in the twelve hours weekly is allowable. The teaching must include instruction in anatomy, physiology, the theory and practice of massage, active and passive movements, bandaging and the use of splints, and such nursing details as are required of the masseuse. Candidates should have a practical acquaintance with the aims and objects to be achieved by massage and a knowledge of the reasons for which treatment is ordered in various conditions.

In the case of hospitals training their own nurses in massage, some reduction in the weekly time-table of instruction may be allowed in consideration of the study already given to physiology, anatomy, and the care of patients, but the Incorporated Society would point out that an equivalent amount of instruction is advisable to enable candidates to pass the examination.

The questions asked at the written examination were as follows :—

### THEORY OF MASSAGE PAPER. (Three hours.)

1. What are your aims in applying: (*a*) pétrissage; (*b*) tapotement?
2. What precautions would you take in treating: (*a*) a case of recently displaced semilunar cartilage; (*b*) a patient with hemiplegia one month after seizure?
3. State how you would treat: (*a*) a compound fracture of the lower third of shaft of humerus beginning on the third day after injury; (*b*) a case of congested liver.
4. How would you prepare and apply a hot fomentation? What points require attention in renewing it?
5. A bullet has severed the ulnar nerve at the elbow. State what results you think are likely to occur and what your treatment would be.
6. To what *reasons* do you attribute the necessity for the rules of the Incorporated Society of Trained Masseuses which you have signed?

### ANATOMY PAPER.

1. Compare the parts of a lumbar vertebra with the corresponding parts of a dorsal and of a cervical vertebra.
2. At what joint or joints does rotation of the head occur? What muscles are concerned in rotating the head? What is the nerve supply of these muscles?
3. What is a "sympathetic" or "visceral" nerve? Describe the course of the vagus in the neck, and name the branches it gives off in the cervical part of its course.
4. Trace the course and termination of the radial artery.
5. Describe the position of the right kidney and trace the course of the urine from the kidney to the internal urinary meatus.
6. What is a superficial fascia? What is a deep fascia? Illustrate your answer by reference to the fascia of the forearm.

(Two and a half hours allowed. Use diagrams freely.)

Owing to the increased number of candidates, the Incorporated Society of Trained Masseuses are now holding examinations at frequent intervals, and the next examination, to be held in June, will deal with over 400 candidates.

